# Serum Procalcitonin Levels in Postoperative Monitoring of Diabetic Patients with Posterior Lumber Vertebral Stabilization Surgery: A Prospective Comparative Study

**DOI:** 10.5152/TJAR.2022.21272

**Published:** 2022-06-01

**Authors:** İbrahim Hakkı Tör, Sedef Gülçin Ural

**Affiliations:** Department of Anaesthesiology and Reanimation, Health Sciences University Erzurum Regional Training and Research Hospital, Erzurum, Turkey

**Keywords:** Instrumentation, posterior lumbar surgery, procalcitonin, surgical site infection

## Abstract

**Objective:**

To compare the serum procalcitonin levels and other infection markers released in response to the inflammatory response that develops secondary to the operation in patients with or without type 2 diabetes mellitus who underwent spinal instrumentation.

**Methods:**

Fifty patients, who belonged to the American Society of Anesthesiologist I-II, were between 18 and 65 years of age, and who had planned for posterior spinal instrumentation surgery were grouped into 2 as group I (n = 25) type 2 diabetic patients (group DM) and group II (n = 25) non-diabetic patients (group non-DM). On the operation day, preoperatively (*T*_0_), 5 minutes after intraoperative instrument placement (T_1_), on postoperative 24th hour (T_2_), 48th hour (T_3_), 3rd day (T_4_), 5th day (T_5_), 7th day (T_6_), 10th day (T_7_), and 15th day (T_8_), serum samples were obtained from the patients for the evaluation of procalcitonin, C-reactive protein, erythrocyte sedimentation rate, and neutrophil values.

**Results:**

Procalcitonin levels were higher in the diabetic patient group at all time points (*P* < .01); C-reactive protein levels were higher in T_1_, T_2_, and T_5_ in the diabetic patient group (*P* < .05). There was no difference in erythrocyte sedimentation rate or neutrophil counts between the groups (*P* > .05). When the alterations in procalcitonin levels were compared between diabetic and non-diabetic groups, in diabetic patients, there were significantly higher increases in the first 6 timelines (*P* < .05).

**Conclusion:**

In diabetic patients, the procalcitonin levels were significantly higher at all time points, predicting an augmented bacterial infection in those patients compared with the non-diabetic patients.

**Main Points**

- In the postoperative period, since wound infection is associated with a high risk of morbidity and mortality, early determination of risk factors and prompt treatment are essential.
- In diabetic patients, procalcitonin levels were significantly higher at all time points, predicting an augmented infection/inflammation in those patients compared with the non-diabetic patients.
- Considering the outcomes of postoperative infections, procalcitonin may be suggested as a marker for the follow-up and treatment of the diabetic patient group with a high probability of infection.

## Introduction

Posterior lumbar vertebral stabilization is an important and commonly performed surgical method in lumbar surgery.^1^ However, surgical wound infection is one of the most severe complications in the early postoperative period after spinal surgery with an incidence of 0.7%-12%.^2-4^ Since wound infection is associated with a high risk of morbidity and mortality, early determination of risk factors and prompt treatment are essential. Diabetes mellitus (DM) is known as an independent risk factor for postoperative incision infection and increases the risk 2-5 times in diabetic patients compared with non-diabetic patients.^1^

The inflammatory response develops as a reaction to infections. Acute phase reactants such as C-reactive protein (CRP) and erythrocyte sedimentation rate (ESR), which are used as the markers of the systemic inflammatory response and are not specific for infection. Therefore, more specific markers are required in the detection and follow-up of infections.^5^ Procalcitonin (PCT) is a 116 amino acid glycopeptide produced by C cells of the thyroid gland under normal conditions and is a precursor to calcitonin.^6,7^ It is known that serum PCT levels increase significantly in cases of sepsis and severe invasive bacterial infections and decrease rapidly with appropriate antibiotic treatment. However, the PCT level does not change in severe viral infections and/or other inflammatory diseases. It is thought that PCT, which is found in high concentrations in severe bacterial infections, has a functional meaning in immune defense. A study performed on in vitro human lymphocytes observed that PCT inhibits arachidonic acid product prostaglandin and thromboxane production in lymphocytes.^8^ It is believed that this inhibition is similar to the effect of nonsteroidal anti-inflammatory analgesics or aspirin, that is, it occurs as a result of the inhibition of cyclooxygenase activity. 

The primary aim of this study is to evaluate the per-operative PCT levels in patients undergoing spinal instrumentation and compare the alterations in PCT levels in diabetic and non-diabetic patients. The secondary aim of the study is to determine whether there is a linear relationship between the PCT levels or other infection parameters and the presence of diabetes.

## Methods

The Clinical Research Ethics Committee approved the study at the Health Sciences University Erzurum Regional Training and Research Hospital (January 20, 2020-2020/02-16). Fifty patients who belonged to the American Society of Anesthesiologist I-II, who were between 18 and 65 years of age, and who were scheduled for posterior spinal instrumentation surgery were included in the study and grouped into 2 as group I (n = 25) type 2 diabetic patients (DM group) and group II (n = 25) non-diabetic patients (non-DM group). In the DM group, only the patients who were taking oral anti-diabetic medications and who were not under insulin treatment were included. Patients with type 1 diabetes, with hemoglobin A1C (HbA1C) >6.5%, having surgery history within the last 3 months, in pregnancy or lactation period, with the urinary tract, lung, or skin infection, with chronic rheumatic disease at the time of application, with a diagnosis of malignancy, with advanced liver and/or kidney dysfunction (GFR <30 mL min-1), who were recently diagnosed with sepsis, and who received immunosuppressive therapy were excluded from the study. The patients were included consecutively. 

On the operation day, preoperatively (T0), 5 minutes after intraoperative instrument placement (T1), on postoperative 24th hour (T2), 48th hour (T3), 3rd day (T4), 5th day (T5), 7th day (T6), 10th day (T7), and 15th day (T8), serum samples were obtained from the patients for the evaluation of PCT, CRP, ESR, and neutrophil levels. Postoperative patient follow-up was continued in the postoperative control unit on the first day, and the follow-up was done in the neurosurgery clinic on the following days.

The patients who were discharged were contacted by phone and told to come to their controls on the 10th and 15th days. In this way, necessary blood samples were taken and assay controls were performed.

Normal PCT values were considered as 0.021-0.500 ng mL-1 (Archem^®^, İstanbul, TURKEY). Normal CRP values in healthy individuals are on average 0.8 mg L-1 and are found below 3 mg L-1 in the majority of the population (Archem^®^, İstanbul, TURKEY).

### Sample Size Calculation and Power Analysis

The sample size calculation was done with the G*Power version 3.1.9.4 (Kiel University, Kiel, Germany) software. In the power analysis performed with the PCT values measured at 72 hours, it was determined that the effect size was 0.95 at the 95% CI and 0.95 at the power significance level. The sample size was calculated as 25 patients for each group and 50 patients in total. This result indicated that the study sample size was sufficient.

### Statistical Analysis

Statistical analysis was performed with International Business Machines Statistical Package for the Social Sciences software v22.0 (IBM Corp.; Armonk, NY, USA) package. The normality distribution of variables was checked with the Shapiro–Wilk and histogram tests. Descriptive data were expressed as mean ± standard deviation (SD). Normally distributed data comprising continuous variables were analyzed using the Student’s *t*-test. Variables that are not normally distributed comprising continuous variables were analyzed using the Mann–Whitney *U* test. Categorical variables were analyzed using the chi-square test. 

## Results

A total of 50 patients were included in this study (25 patients in each group). There was no statistically significant difference between the groups in terms of demographic characteristics (*P* > .05) (Table 1).

When the 2 groups were compared in terms of PCT levels at all times, the measured PCT values were higher in the DM group compared to the non-DM group, and there was a statistically significant difference in the PCT level (*P* < .05) (Table 2). 

When the 2 groups were compared in terms of CRP parameters, a statistically significant difference was observed between DM and non-DM groups in T1, T4, and T5 (*P*  = .048, *P*  = .008, and *P*  = .015, respectively) (Table 2). When the 2 groups were compared in terms of ESR or neutrophil count at all measurement times, there was no statistically significant difference between the groups (*P* > .05) (Table 2).

**Figure 1.**
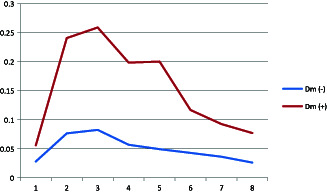
Alterations in procalcitonin levels in time in diabetic and-non diabetic groups.

We also evaluated the alterations in PCT levels in time, in diabetic and-non diabetic groups (Figure 1). When the alterations in PCT levels were compared between diabetic and non-diabetic groups, in diabetic patients, there were significantly higher increases in the first 6 timelines (Table 3). 

## Discussion

The results of the study showed that PCT levels were higher during the perioperative period in diabetic patients than in non-diabetic patients during posterior spinal instrument surgery. Although the diabetic patients were all having HbA1c <6.5 before the operation and all were having regulated blood glucose levels, the presence of DM was associated with increased PCT levels. Moreover, the increases in PCT levels were significantly higher in diabetic patients compared with the non-diabetic patients, which is suggested as a marker of infection/inflammation. In the light of these data, we can suggest that, due to the augmented inflammation or infection in diabetic patients, during the peroperative period, we must be cautious about the infectious complications. These results also give us important data that the perioperative course of PCT and CRP levels is different in patients with non-insulin dependent DM as compared to patients without NIDDM. And the difference in PCT between the 2 study groups at baseline before surgery might suggest that PCT values are different in patients with NIDDM even without surgical trauma. The current study might be used as a pilot for a much larger study investigating this hypothesis and defining normal values of patients with NIDDM.

Despite modern aseptic procedures and surgery techniques, patients are still susceptible to postoperative infections. While the risk of infection in discectomy operations is associated with less than 1%, this ratio is associated with 1%-5% in non-instrumentation spinal fusion surgeries and 6% or more in spinal fusion operations with instrumentation. This risk is even higher in the diabetic patient group.^9^ Diabetes is thought to be a risk factor for surgical site infections. The presence of microangiopathies and neuropathies associated with many systemic complications of DM also increases the relationship with surgical site infections. Precise prediction of the presence and severity of infection in diabetic patients plays an essential role in infection control and prognosis. Although bacterial culture is a gold standard in diagnosing infection, the extended test time and low sensitivity limit its clinical use. Therefore, it is more common to use serum markers such as CRP and neutrophil count for infection prediction in the perioperative period.^10,11^

Despite the prophylaxis applied in instrumentation surgeries, the frequency of serious infections is 2.2%-8.5%.^12^ It is often challenging to diagnose postoperative spinal infection unless clinical signs become evident. Although there are inflammatory markers such as CRP, WBC count, ESR, and body temperature that can be easily obtained, their specificity is not high. In our study, we observed that PCT levels increased more in the diabetic group at all measurement times. We also observed that CRP, ESR, and neutrophil values were also higher in the diabetic group compared to the non-diabetic group.

Under normal conditions, the half-life of PCT secreted from the thyroid gland’s parafollicular C cells is 25-30 hours.^9^ During infection, PCT is ectopically secreted into the peripheral blood circulation by the liver’s neuroendocrine cells, peripheral blood monocytes, macrophages, spleen, lung, small intestine, and kidneys. Levels rapidly increase as the bacterial infection progresses but remain low during viral infections and nonspecific inflammatory diseases such as ulcerative colitis.^13^ In many studies in the literature, it has been stated that PCT is used safely to exclude bacterial infection and to prevent inappropriate antibiotic treatments when compared with other traditional serum markers such as CRP and neutrophil count.^14,15^ Procalcitonin, which is barely detectable in healthy individuals, begins to rise within 2 hours in the presence of widespread bacterial infection, rises rapidly within 6 hours, and peaks in 24 hours.^16^ Therefore, PCT is an essential parameter in the early diagnosis of sepsis.^17^

Procalcitonin is reported to be superior to other infection markers in diagnosing acute bacterial infections.^18,19^ In a retrospective study by Zhang et al,^20^ infection markers were evaluated in terms of perioperative pneumonia, urinary infections, and superficial surgical site infections in 500 patients who underwent hip and knee arthroplasty and it has been reported that PCT has a high sensitivity (96%) and specificity (100%) in the diagnosis of bacterial infection. In the study conducted by Abu Elyazed et al,^21^ PCT and CRP were compared in the early diagnosis of hospital-acquired pneumonia after abdominal surgery; PCT was determined to have higher sensitivity and specificity on postoperative day 2 compared to CRP levels (84% and 72% vs 70% and 60%, respectively). Nie et al^22^ also argued that PCT is superior to CRP in the early diagnosis of postoperative infectious complications in traumatic spinal cord injuries and had a significant role in establishing an effective antibiotic treatment regimen in the postoperative period. In our study, we observed that PCT increased before other infection markers as an infection parameter in both the diabetic and the non-diabetic group. We also determined a higher increase in PCT levels in diabetic patients, which may be suggested as a predictor or marker of bacterial infection after posterior lumber vertebral stabilization surgery. 

Fever above 38°C, which can be seen in the early postoperative period commonly, is caused by the inflammatory stimulus of operation and mostly resolves spontaneously. However, this fever may also be a harbinger of serious complications such as surgical site infection, drug fever, or deep vein thrombosis. Among the laboratory parameters used for the identification of these conditions, PCT, CRP, ESR, and neutrophil count are used. Erythrocyte sedimentation rate is a non-specific marker of inflammation with limited clinical use. Its usefulness as a screening test is limited by its low sensitivity and specificity in postoperative patients.^9^ C-reactive protein is an acute-phase protein synthesized from hepatocytes in response to inflammation and characterized by high sensitivity and rapid response formation.^23^ After surgery, CRP levels rise rapidly on the 3rd postoperative day and regress to baseline levels between the 10th and 14th postoperative days.^24^ However, non-infectious factors such as the operation site and surgery type also affect the CRP levels.^25,26^ In the study conducted by Iwata et al,^24^ it was reported that the CRP level measurement on the seventh postoperative day in the diagnosis and follow-up of surgical site infections in patients who underwent instrumentation surgery had high sensitivity and specificity in terms of being an infection marker. In our study, we observed that CRP levels increased from the postoperative 24th hour, peaked on the postoperative 3rd day, and then decreased, which supports the studies mentioned. These results we obtained in the study are compatible with the literature.

Our study has some limitations. Firstly, during the period after discharge outside the hospital, patients may have changed their biomarker levels with some uncontrollable factor. Secondly, we believe that the results would be more reliable if the study had a double-blind design. 

## CONCLUSIONS

In diabetic patients, the PCT levels were significantly higher at all time points, predicting an augmented infection/inflammation in those patients compared with the non-diabetic patients. In the light of these data, we can suggest that, due to the augmented inflammation or infection in diabetic patients, during the peroperative period, we must be cautious about the infectious complications. And the difference in PCT between the 2 study groups at baseline before surgery might suggest that PCT values are different in patients with NIDDM even without surgical trauma. The current study might be used as a pilot for a much larger study investigating this hypothesis and defining normal values of patients with NIDDM.

## Figures and Tables

**Table 1.** Demographic Data and the Number of Instrumentation Level Between Group DM and Group Non-DM

	Group Non-DM (n = 25)	Group DM (n = 25)	*P*
Age (years)	48.88 ± 11.33	53.68 ± 10.26	.123^a^
Gender (M/F)	9/16	10/15	1.000^b^
ASA (I/II)	14/11	9/16	.256^b^
Number of instrumentation level	6.48±1.75	6.96±1.64	.324^a^
BMI	28.52±4.24	29.48±3.77	.573^a^
Smoking (no/yes)	14/11	16/9	.773^b^

Values are expressed as mean ± standard deviation or number.

M, male; F, female; ASA, American Society of Anesthesiologists; DM, diabetes mellitus; BMI, body mass index.

^a^Student’s *t*-test between groups; ^b^Chi-square test between groups.

**Table 2.** The Comparison of Procalcitonin, CRP, Sedimentation, and Neutrophil values Between Group DM and Group non-DM

	Group non-DM (n = 25) Median [25%-75%]	Group DM (n = 25) Median [25%-75%]	*P*
**Procalcitonin (ng/L)**			
T_0_	0.019 [0.019-0.040]	0.041[0.025-0.062]	**.003**
T_1_	0.022 [0.019-0.047]	0.055 [0.040-0.080]	**.001**
T_2_	0.047 [0.030-0.118]	0.120[0.071-0.188]	**.002**
T_3_	0.063 [0.031-0.109]	0.144 [0.094-0.220]	**.001**
T_4_	0.051 [0.025-0.072]	0.122 [0.075-0.188]	**.001**
T_5_	0.035 [0.024-0.082]	0.100 [0.067-0.280]	**.001**
T_6_	0.039 [0.027-0.060]	0.088 [0.058-0.138]	**.001**
T_7_	0.028 [0.019-0.048]	0.067 [0.047-0.092]	**.001**
T_8_	0.019 [0.019-0.027]	0.047 [0.034-0.084]	**.001**
**CRP (mg L-1)**			
T_0_	3.12 [1.90-6.00]	5.02 [2.97-8.96]	.054
T_1_	2.86 [1.89-4.38]	4.90 [2.92-10.10]	**.042**
T_2_	54.10 [18.80-77.50]	66.20 [37.60-90.40]	.055
T_3_	82.50 [51.40-111.00]	107.00 [79.80-141.00]	.068
T_4_	67.20 [51.40-100.00]	108.00 [71.10-136.00]	**.008**
T_5_	54.90 [29.50-91.50]	80.00 [57.90-117.00]	**.015**
T_6_	47.00 [30.30-90.20]	71.80 [49.30-108.50]	.090
T_7_	38.50 [18.40-68.00]	52.60 [35.40-75.40]	.097
T_8_	27.00 [11.60-35.00]	28.40 [17.80-70.50]	.204
**Sedimentation (mm h-1)**			
T_0_	60.2 [54.6-63.3]	56.5 [52.2-71.2]	.336
T_1_	61.2 [52.9-66.6]	58.2 [52.3-72.2]	.553
T_2_	77.6 [75.1-80.3]	78.0 [73.9-83.2]	.466
T_3_	73.8 [67.0-79.6]	74.8 [70.9-80.2]	.232
T_4_	66.9 [60.2-74.4]	71.8 [66.6-75.0]	.087
T_5_	69.4 [64.9-72.9]	69.6 [62.3-72.9]	.061
T_6_	68.4 [64.4-70.9]	64.2 [61.8-69.2]	.478
T_7_	62.7 [53.2-70.3]	66.3 [61.3-72.8]	.473
T_8_	61.8 [51.2-67.5]	62.1 [58.8- 69.9]	.432
**Neutrophil (%)**			
T_0_	60.2 [54.6-63.3]	56.5 [52.2-71.2]	.720
T_1_	61.2 [52.9-66.6]	58.2 [52.3-72.2]	.900
T_2_	77.6 [75.1-80.3]	78.0 [73.9-83.2]	.786
T_3_	73.8 [67.0-79.6]	74.8 [70.9-80.2]	.438
T_4_	66.9 [60.2-74.4]	71.8 [66.6-75.0]	.233
T_5_	69.4 [64.9-72.9]	69.6 [62.3-72.9]	.786
T_6_	68.4 [64.4-70.9]	64.2 [61.8-69.2]	.184
T_7_	63.7 [53.2-70.3]	66.3 [61.3-72.8]	.144
T_8_	61.8 [51.2-67.5]	62.1 [58.8-69.9]	.162

Mann–Whitney *U* test was performed for the comparison between groups.

Preoperative operation day (T0), 5 minutes after intraoperative instrument placement (T1), postoperative 24th hour (T2), postoperative 48th hour (T3), postoperative third day (T4), postoperative fifth day (T5), postoperative seventh day (T6), postoperative 10th day (T7), postoperative 15th day (T8).

**Table 3.** Changes in Procalcitonin Levels in Time in Diabetic and Non-diabetic Groups

Alterations in PCT levels	Non-diabetic Group (n = 25)	Diabetic Group (n = 25)	*P*
Δ1 (T1-T0)	0.0045 ± 0.009	0.0167 ± 0.027	**.04**
Δ2 (T2-T0)	0.048 ± 0.068	0.184 ± 0.351	**.04**
Δ3 (T3-T0)	0.054 ± 0.064	0.203 ± 0.311	**.02**
Δ4 (T4-T0)	0.028 ± 0.040	0.142 ± 0.189	**.006**
Δ5 (T5-T0)	0.0212 ± 0.024	0.144 ± 0.199	**.004**
Δ6 (T6-T0)	0.0148 ± 0.0148	0.0608 ± 0.083	**.011**
Δ7 (T7-T0)	0.0082 ± 0.0187	0.0366 ± 0.091	.21
Δ8 (T8-T0)	−0.0019 ± 0.012	0.021 ± 0.092	.16

Preoperative operation day (T0), 5 minutes after intraoperative instrument placement (T1), postoperative 24th hour (T2), postoperative 48th hour (T3), postoperative third day (T4), postoperative fifth day (T5), postoperative seventh day (T6), postoperative 10th day (T7), postoperative 15th day (T8).
